# Dietary Antigens Induce Germinal Center Responses in Peyer's Patches and Antigen-Specific IgA Production

**DOI:** 10.3389/fimmu.2019.02432

**Published:** 2019-10-15

**Authors:** Satoko Hara, Takaharu Sasaki, Naoko Satoh-Takayama, Takashi Kanaya, Tamotsu Kato, Yui Takikawa, Masumi Takahashi, Naoko Tachibana, Kwang Soon Kim, Charles D. Surh, Hiroshi Ohno

**Affiliations:** ^1^Laboratory for Intestinal Ecosystem, RIKEN Center for Integrative Medical Sciences, Yokohama, Japan; ^2^Division of Immunobiology, Department of Medical Life Science, Graduate School of Medical Life Science, Yokohama City University, Yokohama, Japan; ^3^Institute for Basic Science (IBS), Academy of Immunology and Microbiology, Pohang, South Korea; ^4^Kanagawa Institute of Industrial Science and Technology, Kanagawa, Japan

**Keywords:** IgA, dietary antigens, Peyer's patches, mucosal immunology, germinal center (GC) reaction

## Abstract

The primary induction sites for intestinal IgA are the gut-associated lymphoid tissues (GALT), such as Peyer's patches (PPs) and isolated lymphoid follicles (ILFs). The commensal microbiota is known to contribute to IgA production in the gut; however, the role of dietary antigens in IgA production is poorly understood. To understand the effect of dietary antigens on IgA production, post-weaning mice were maintained on an elemental diet without any large immunogenic molecules. We found that dietary antigens contribute to IgA production in PPs through induction of follicular helper T cells and germinal center B cells. The role of dietary antigens in the PP responses was further confirmed by adding bovine serum albumin (BSA) into the elemental diet. Although dietary antigens are important for PP responses, they have fewer effects than the microbiota on the development and maturation of ILFs. Furthermore, we demonstrated that dietary antigens are essential for a normal antigen-specific IgA response to *Salmonella typhi* serovar Typhimurium infection. These results provide new insights into the role of dietary antigens in the regulation of mucosal immune responses.

## Introduction

The microbiota and diet are the major sources of exposure to foreign antigens and therefore are expected to influence the properties of immune cells, and contribute to various aspects of immune responses. It has been well-known, for example, that commensal bacteria induce the production of IgA in the gut and the expansion of CD4^+^ T cells such as T-helper-17 (Th17) or regulatory T cells (1–3). A recent study has demonstrated that dietary antigens are essential for the induction of retinoic acid receptor-related orphan receptor gamma (RORγt)-negative peripheral regulatory T cells in the small intestine (SI) (4). When germ-free (GF) mice were kept on an antigen-free (AF) diet, a chemically defined elemental diet without any large potentially immunogenic molecules (GF-AF), peripheral regulatory T (Treg) cells, and serum IgA were significantly decreased compared with GF mice that were raised on a normal diet including large molecules. However, the role of dietary antigens in mucosal immune responses is still unknown. Since IgA is the most abundant Ig in the gut (5), the decreased amount of serum IgA in GF-AF mice raises the possibility that intestinal IgA secretion is also decreased in GF-AF mice.

The intestinal IgA response is evoked in gut-associated lymphoid tissues (GALT), such as isolated lymphoid follicles (ILFs), Peyer's patches (PPs), and mesenteric lymph nodes (MLNs) (6, 7). Among them, PPs are a crucial site for the development of antigen-specific IgA^+^ B cells (8). Generation of PPs occurs during embryogenesis, and the number of PPs is 6–12 per mouse (9, 10). PPs harbor organized follicular structures and the follicles contain germinal centers (GCs) where somatic hypermutation (SHM) of Ig variable region genes and class switching from IgM to IgA are induced and B cells with high-affinity B-cell receptors are selected (11). IgA^+^ B cells generated in GC of PPs migrate into the lamina propria (LP) and differentiate into IgA^+^ plasma cells. The IgA produced by these plasma cells is transcytosed by the polymeric immunoglobulin receptor (pIgR) expressed on intestinal epithelial cells and secreted into the gut lumen as secretory IgA (12). GC B cells interact with follicular helper T (Tfh) cells to promote the expansion of the GC B cells with a high affinity for the antigen and the apoptosis of those with low affinity or autoreactivity (13, 14). GC B cells with a high affinity for antigen can differentiate into plasma cells by forming more stable contacts with Tfh cells than the low affinity GC B cells (15). Tfh cells are characterized by the high expression of CXCR5 and PD-1 (16, 17). MLNs also harbor GC B cells and Tfh cells to produce IgA. It has been reported that the microbiota affects the properties of GC B cells and Tfh cells in GALT; however, it is unclear whether dietary antigens impact on these processes.

ILFs harbor a single B-cell follicle with a GC which, along with PPs and MLNs, is involved in IgA production (18, 19). Approximately 100–200 ILFs develop in the SI and 50–100 in the large intestine (LI) of mature mice (18). In contrast to PPs, ILFs develop postnatally in mice, and their distribution in the SI has a regional dependency (20); the number of ILFs is more abundant in distal than in proximal SI (20). This strongly suggests that the microbiota contributes to the development of ILFs, because commensal bacteria are much more abundant in the distal than in the proximal SI. On the other hand, the microbiota represses the development of colonic ILFs (20). However, the role of dietary antigens in the development of ILFs is also poorly understood.

In this study, we found that GF-AF mice had a significant decrease in IgA production in the SI, which strongly correlated with decreased numbers of GC B cells and Tfh cells in the GALT. In addition, we found that GF-AF mice had a decreased antigen-specific intestinal IgA response and were more susceptible than GF mice to infection with the intestinal pathogen *Salmonella typhi* serovar Typhimurium.

## Results

### Dietary Antigens Contribute to IgA Production in SI but Not in LI

To assess the roles of dietary antigens in the intestinal immune system, we prepared an antigen-free (AF) diet and established GF-AF conditions in isolators (4, 21, 22). Serum IgA and the total number of leukocytes obtained from SI-LP and MLNs were decreased in GF-AF mice compared to GF and SPF mice (Supplemental Figures 1A–C). By contrast, GF-AF, GF, and SPF mice all harbor a comparable number of leukocytes in the spleen and the LI-LP (Supplemental Figures 1D,E). These results indicate that our GF-AF mice have a similar phenotype to those described in a previous report (4).

**Figure 1:**
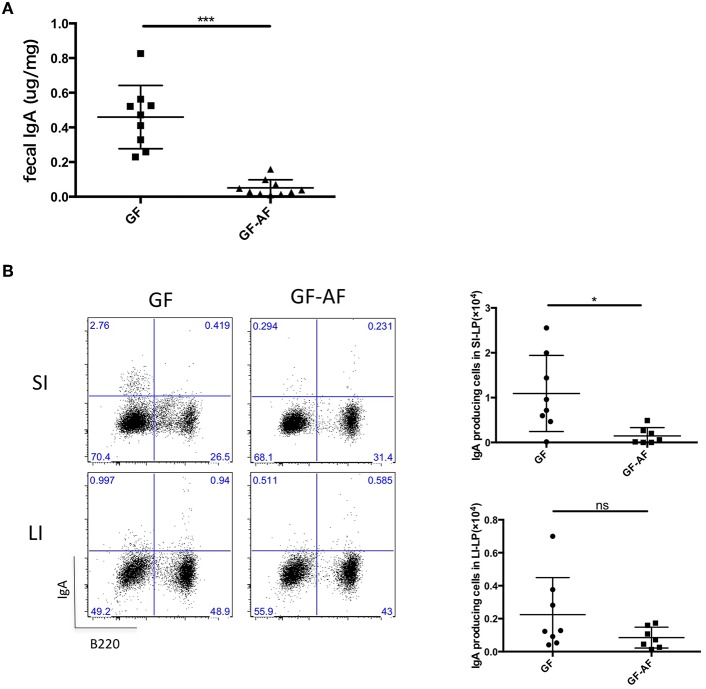
The elimination of dietary antigens decreases IgA production in the SI. **(A)** IgA concentration in feces from GF (*n* = 9) and GF-AF mice (*n* = 10). **(B)** Representative flow cytometry plots of IgA vs. B220 on CD3^−^ lymphocytes of SI-LP and LI-LP of GF and GF-AF mice (left), and the absolute numbers of B220^−^IgA^+^ IgA-producing plasma cells (right). Data are pooled from at least three independent experiments and are presented as mean ± SD. Welch's *t*-test was used for statistical analysis. *p < 0.05, ***p < 0.001.

We next investigated whether dietary antigens affected IgA production in the gut. SPF-AF mice showed reduced fecal IgA and IgA producing cells compared to SPF mice (Supplemental Figures 2A,B) and they also had a different fecal microbial composition compared to that in SPF mice at phylum and genus levels (Supplemental Figures 2C,D). A previous study has reported that IgA is induced by the colonization of the microbiota (23, 24), and we also observed a reduction in fecal IgA of GF mice (Supplemental Figure 1F) in keeping with the decreased number of IgA-producing plasma cells in the small and large intestines (Supplemental Figures 1G,H). Thus, we compared the difference between GF and GF-AF mice. Compared to GF mice, fecal IgA and the number of IgA-producing plasma cells (B220^−^IgA^+^) in the SI-LP from GF-AF mice were decreased; by contrast, there was no significant difference between GF and GF-AF mice in the number of IgA plasma cells in the LI-LP (Figures 1A,B). These results suggest that dietary antigens contribute to the generation of IgA plasma cells in the SI but not so much in the LI. This seems reasonable, since most dietary antigens are digested in the SI and thus are less exposed to the LI.

### Dietary Antigens Increase the Size and Number of Lymphocytes in PPs

**Figure 2:**
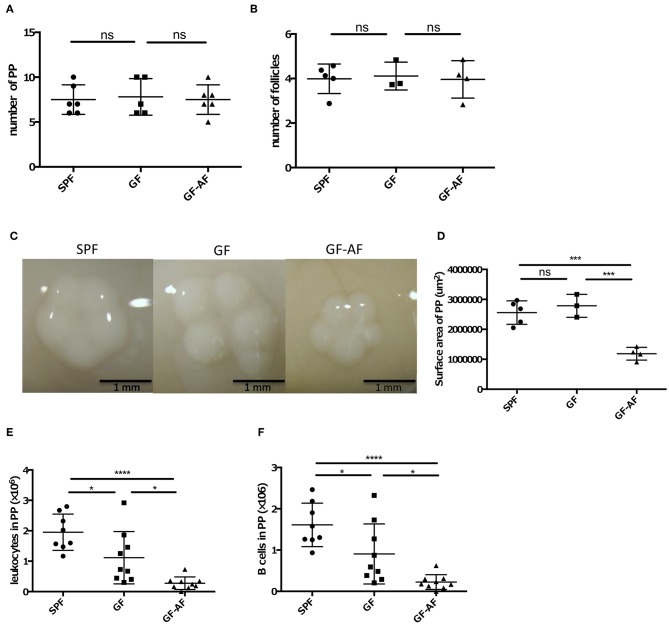
The PPs in GF-AF mice are smaller than in SPF and GF mice. **(A)** The numbers of PP in SPF (*n* = 6), GF (*n* = 5), and GF-AF (*n* = 6) mice. Data are pooled from at least two independent experiments. **(B)** The average number of follicles per PP of SPF (*n* = 5), GF (*n* = 3), and GF-AF (*n* = 4) mice. Data are pooled from at least two independent experiments. **(C)** Representative images of PPs from SPF, GF and GF-AF mice. Bar, 1 mm. **(D)** The area of each PP was measured. Each point shows the average PP circumferences from one mouse. SPF (*n* = 5), GF (*n* = 3), and GF-AF (*n* = 4) mice were examined. Data are pooled from at least two independent experiments. **(E)** The number of leukocytes in PP of SPF (*n* = 8), GF (*n* = 9), and GF-AF (*n* = 9) mice. Data are pooled from at least three independent experiments. **(F)** The number of B cells (CD3^−^ CD19^+^ lymphocytes) in PP of SPF (*n* = 8), GF (*n* = 9), and GF-AF (*n* = 9) mice. Data are pooled from at least three independent experiments. Mean ± SD are shown. One-way ANOVA with Tukey's *post-hoc* test was performed for statistical analysis. *p < 0.05, ***p < 0.001, ****p < 0.0001.

We next examined how dietary antigens are involved in the production of IgA in the SI. As described above, PPs are an important site for the production of IgA^+^ B cells. We therefore evaluated the roles of dietary antigens in the development of PPs. GF-AF, GF, and SPF mice harbored comparable numbers of PPs and follicles in each PP (Figures 2A,B), indicating that neither dietary antigens nor the microbiota are involved in the generation of PPs. On the other hand, the size of PPs in GF-AF mice was significantly smaller than that of SPF and GF mice (Figures 2C,D). Consistent with the decreased size of PPs, the number of leukocytes and B cells obtained from PPs was decreased in GF-AF mice compared to SPF and GF mice (Figures 2E,F). Collectively, it appears that dietary antigens contribute to the recruitment of immune cells into follicles in PPs, but not to PP generation.

### Dietary Antigens Induce Tfh Cells and GC B Cells in PP

**Figure 3:**
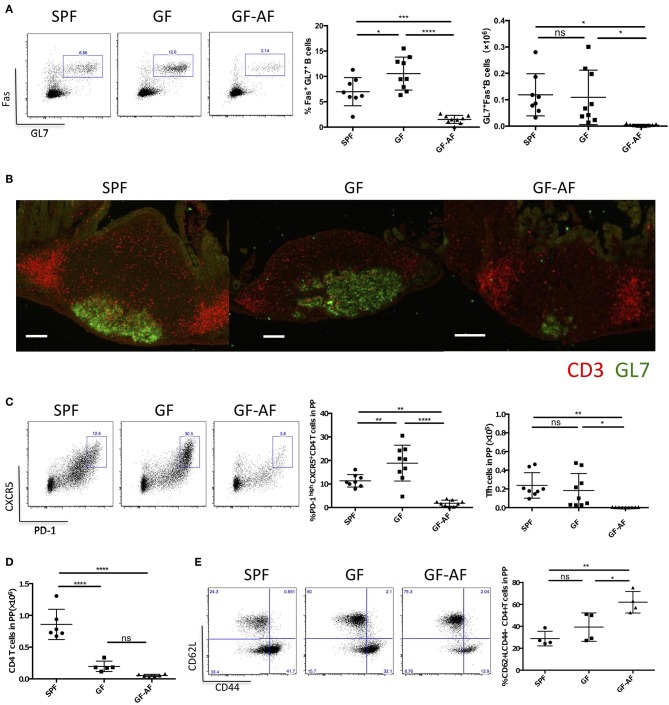
The number of GC B cells and Tfh cells in PPs is dramatically decreased in GF-AF mice. **(A)** Representative flow cytometry plots of Fas vs. GL7 expression by PP B220^+^ CD19^+^ lymphocytes from SPF, GF, and GF-AF mice. The numbers in the boxes indicate percentage of Fas^+^ GL7^+^ cells in the gate (left). Percentages and absolute cell numbers of Fas^+^ GL7^+^ B cells in PPs are shown (right) (SPF; *n* = 8, GF; *n* = 9, and GF-AF; *n* = 9). Data are pooled from at least three independent experiments. **(B)** Vertical PP sections were stained with GL7 (green) and CD3 (red) antibodies to detect GC B cells and T cells (*n* = 3). Bars, 100 μm. This image is representative of two independent experiments. **(C)** Representative flow cytometry plots of CXCR5 vs. PD-1 expression by CD19^−^ CD3^+^ CD4^+^ lymphocytes from PPs of SPF, GF, and GF-AF mice. The numbers in the boxes indicate percentage of CXCR5^+^ PD-1^+^ cells in the gate (left). Percentages and absolute cell numbers of CXCR5^+^ PD-1^+^ cells among CD19^−^ CD3^+^ CD4^+^ lymphocytes of PP are shown (right). (SPF; *n* = 8, GF; *n* = 9, and GF-AF; *n* = 9). Data are pooled from at least three independent experiments. **(D)** Absolute number of CD3^+^ CD4^+^ lymphocytes. (SPF; *n* = 6, GF; *n* = 4, and GF-AF; *n* = 6). Data are pooled from at least three independent experiments. **(E)** Representative flow cytometry plots (left) and percentages of CD62L vs. CD44 among CD19^−^ CD3^+^ CD4^+^ lymphocytes from PP of SPF (*n* = 4), GF (*n* = 4), GF-AF (*n* = 4) mice (right). Data are pooled from two independent experiments. Mean ± SD are shown. One-way ANOVA with Tukey's *post-hoc* test was performed for statistical analysis. *p < 0.05, **p < 0.01, ****p < 0.0001.

Since it has been demonstrated that GC B cells and Tfh cells are crucial for the maturation of IgA^+^ B cells in PPs (8), we further characterized these cells in GF-AF mice. The frequency and absolute number of GC B cells (GL7^+^Fas^+^ B cells) among total B cells were markedly decreased in GF-AF mice compared with GF mice, whereas these were almost comparable between GF and SPF mice (Figure 3A). Consistent with the flow cytometry analysis, immunohistochemical analysis revealed a reduction of GL7 expression in PPs of GF-AF mice (Figure 3B). CXCR5^+^PD-1^+^ Tfh cells among CD4^+^ T cells were also drastically decreased in GF-AF mice compared with GF and SPF mice (Figure 3C). The numbers of PP leukocytes and GC B cells were restored by the addition of BSA to the AF diet, further confirming that dietary antigens contribute to the induction of GC B cells in PPs (Supplemental Figures 3A,B).

To more specifically evaluate the contribution of dietary antigens vs. the microbiota to the development of GC B cells and Tfh cells, we employed specific-pathogen free (SPF) mice kept on antigen free diets (SPF-AF). Both GC B cells and Tfh cells in PPs were profoundly decreased in SPF-AF mice compared with SPF mice (Supplemental Figures 3C,D). MLNs in GF-AF mice also had decreased numbers of Tfh and GC B cells in comparison with SPF mice and GF mice (Supplemental Figures 3E,F). Together, these observations suggest that dietary antigens also contribute to the induction of Tfh cells and GC B cells in both PPs and MLNs.

We next examined the effect of dietary antigens on the development of CD4^+^ T cells. Although the absolute number of CD4^+^ T cells in PPs was decreased in GF and GF-AF mice compared with SPF mice, with no significant difference between GF and GF-AF mice (Figure 3D), the frequency of CD4^+^ T cells with naïve phenotype (CD62L^+^CD44^−^) (25) among CD4^+^ T cells was higher in GF-AF mice than in GF mice (Figure 3E). Taken together, dietary antigens seem to contribute to the differentiation of naïve CD4^+^ T cells, probably including Tfh cell differentiation (26).

It has also been reported that Foxp3^+^ T cells and Th17 cells could convert into Tfh cells in PPs (27, 28), and that the loss of Tfh cells in PPs of T-cell-deficient mice could be restored by the adoptive transfer of Foxp3^+^ T cells or Th17 cells. However, it remains controversial whether Foxp3^+^ T cells or Th17 cells are the source of Tfh cells in PPs (27, 28). We found that neuropilin-1^low^ RORγt^−^Foxp3^+^ peripheral Treg cells (Nrp-1^low^RoRγt^−^ Treg cells) showed a trend toward a decrease in PPs of GF-AF mice (Supplemental Figure 4A). On the other hand, the number of Th17 cells was significantly decreased in GF mice compared with SPF mice and no further decrease was observed in GF-AF mice (Supplemental Figure 4B). Thus, dietary antigens contribute to the induction of PP Nrp-1^low^RoRγt^−^ Treg cells as well as Tfh cells, but not Th17 cells.

### Dietary Antigens Affect the Development of ILFs Through Interaction With the Microbiota

**Figure 4:**
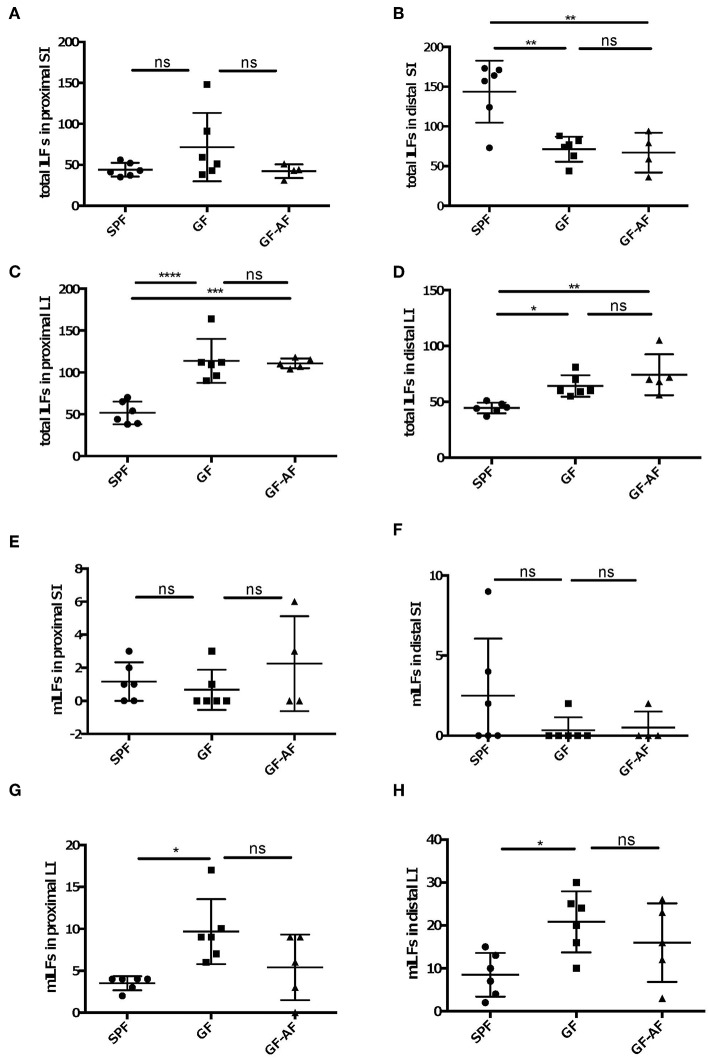
The development and maturation of ILFs are not directly regulated by dietary antigens. **(A–D)** total ILF numbers and **(E–H)** mature ILF numbers in various parts of the intestine of SPF, GF, and GF-AF mice. Maturity of ILFs was evaluated by measuring the B220^+^ area of the ILF and if it was ≥50,000 μm^2^, the ILFs were characterized as “mature.” **(A,E)** proximal SI. **(B,F)** distal SI. **(C,G)** upper half of LI. **(D,H)** lower half of LI of mice. The intestinal regions were defined as described in the Materials and Methods section. Data are pooled from three independent experiments (SI and LI of SPF; *n* = 6, SI and LI of GF; *n* = 6, SI of GF-AF; *n* = 4 and LI of GF-AF; *n* = 5). Mean ± SD are shown. One-way ANOVA with Tukey's *post-hoc* test was performed for statistical analysis. *p < 0.05, **p < 0.01, ***p < 0.001, ****p < 0.0001.

Since ILFs as well as PPs are crucial to induce fecal IgA production, we examined whether dietary antigens affect the development of ILFs. We counted the number of ILFs in the proximal and distal SI, as well as in the LI, since the distribution of ILFs differs region by region in the intestine. The number of ILFs in the distal, but not in the proximal, SI was decreased in both GF and GF-AF mice compared to SPF mice; however, there were no difference in the number of ILFs between GF and GF-AF mice (Figures 4A,B). SPF-AF mice also exhibited a decreased number of ILFs compared to SPF mice in the distal, but not in the proximal, SI (**Supplemental** Figures 5A,B), Taken together, both gut microbiota and dietary antigens seem to be required for the development of SI-ILFs. Alternatively, gut-microbiota-modified food antigens could play a role in this process. Unlike in the SI, the numbers of LI-ILFs significantly increased in GF and GF-AF mice compared to SPF mice (Figures 4C,D), consistent with a previous study (20). However, there was no difference in the number of LI-ILF between GF and GF-AF mice. On the other hand, SPF-AF mice had almost comparable numbers of ILFs in both distal and proximal LI to those of SPF mice (Supplemental Figures 5C,D). Taken together, both gut microbiota and dietary antigens seem to be required for the development of ILFs.

**Figure 5:**
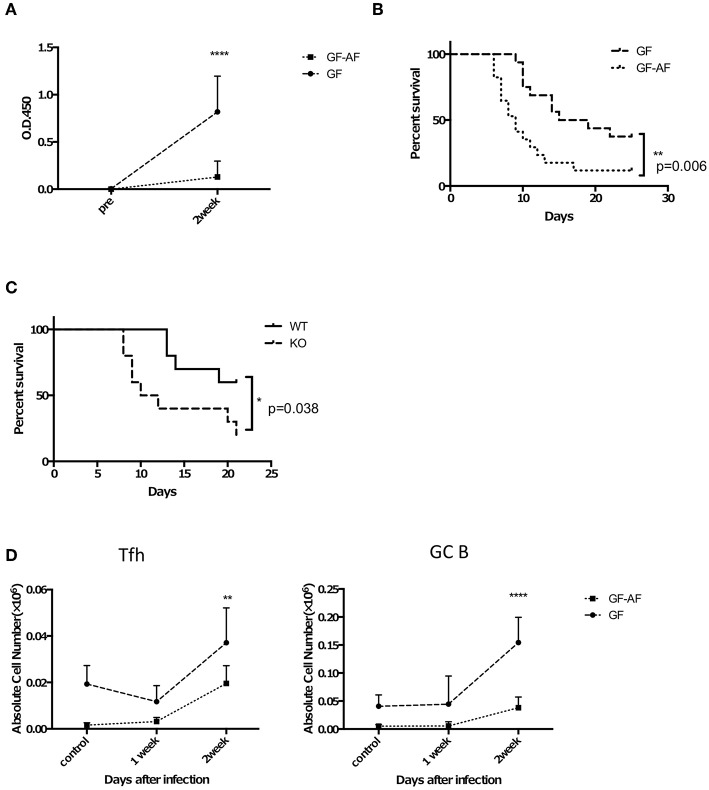
GF-AF mice are more susceptible to *S*. Typhimurium infection than GF mice. Mice were orally infected with attenuated Δ*aroA S*. Typhimurium UF20. Two weeks later, mice were orally challenged with a virulent *S*. Typhimurium, χ3306. **(A)** The amount of *S*. Typhimurium-specific IgA in feces. Feces were collected from GF and GF-A mice before and 2 weeks after infection with UF20. Data are pooled from three independent experiments. **(B)** Kaplan-Meier survival curves after oral administration of χ3306. (GF; *n* = 16, GF-AF; *n* = 17). Data are pooled from three independent experiments. **(C)** AID knockout (*n* = 10) and their littermate wild-type (*n* = 10) mice were infected with *S*. Typhimurium in the same way as **(B)** and the survival curves are shown. Data are pooled from two independent experiments. **(D)** The numbers of Tfh and GF B cells in PP of GF and GF-AF mice before infection and 1 and 2 weeks after infection with UF20 *S*. Typhimurium (*n* = 5 or 6). Data are pooled from two independent experiments. Data are mean ± SD. Log rank test **(B,C)** and two-way ANOVA with Bonferroni's *post-hoc* test **(B–D)** were performed for statistical analysis. *p < 0.05, **p < 0.01, ****p < 0.0001.

We next analyzed the number of mature ILFs (mILFs), which possess a well-organized nodular structure (29). The maturation of ILFs is determined by their size; more than 50,000 μm^2^ aggregates of B220^+^ cells are defined as a mILF (30–32). The number of mILFs in both proximal and distal SI was similar among SPF, GF and GF-AF mice (Figures 4E,F). In addition, there was no significant difference in the number of SI-mILFs between SPF-AF and SPF mice (Supplemental Figures 5E,F). On the other hand, the numbers of mILFs in both proximal and distal LI were significantly increased in GF mice compared with SPF mice, consistent with a previous study (20); however, there was no significant difference in their numbers between GF and GF-AF mice (Figures 4G,H). SPF-AF mice exhibited a significant decrease in the number of mILFs only in the proximal LI (Supplemental Figures 5G,H), suggesting that microbiota exposure to intestinal tissue might be less in the proximal LI of SPF-AF mice compared with SPF mice. Taken together, these results indicate that dietary antigens affect the development and maturation of ILFs, possibly through regulating the composition of the gut microbiota and/or modification by gut microbiota.

### GF-AF Mice Are Susceptible to Oral *Salmonella* Infection

Based on our finding that GF-AF mice showed impaired IgA responses prominently in the SI, we speculated that they would exhibit a defect in antigen-specific IgA production upon infection with a mucosal pathogen, as was seen in a previous study of PP-deficient mice (33). To test this hypothesis, we employed *Salmonella* enterica serovar Typhimurium, which is preferentially taken up into PPs (34). Attenuated *S*. Typhimurium lacking the auxotrophic *aroA* gene (35) were orally gavaged into GF and GF-AF mice in order to assess long-term IgA responses and their feces were collected prior to and 2 weeks after infection. As expected, the amount of *S*. Typhimurium-specific IgA in feces was significantly reduced in GF-AF mice compared with GF mice at 2 weeks after infection (Figure 5A). Consistent with this, GF-AF mice were more susceptible to virulent *S*. Typhimurium than GF mice (Figure 5B). Activation-induced cytidine deaminase (AID)-deficient mice, which completely lack IgA (36), also exhibited increased susceptibility to virulent *S*. Typhimurium infection (Figure 5C), which supports our hypothesis that impaired IgA responses increase mortality after *S*. Typhimurium infection. In addition, there was a decreased expansion of both GC B cells and Tfh cells in PPs from GF-AF mice upon *S*. Typhimurium infection (Figure 5D), suggesting that GC responses constitutively induced by dietary antigens are crucial for induction of IgA responses required for protective immunity against intestinal pathogens.

## Discussion

Involvement of dietary antigens in the PP responses and the overall intestinal immune response against infection have remained poorly understood. Here we demonstrated that dietary antigens affect both PP GC responses and immune responses against *S*. Typhimurium infection, indicating that dietary antigens play an important role in host protection.

In 1970's, some seminal reports were published related to dietary antigens, such as a method for making an “antigen-free” diet, or the effect of dietary antigens on serum immunoglobulin levels (21). Although these studies were informative in terms of systemic immune responses, until recently, the role of dietary antigens in immune responses in the gut has been poorly understood, although the involvement of microbiota in the induction and regulation of the immune responses is well-known (1–3). It has recently been demonstrated that dietary antigens contribute to the induction of Treg cells in the SI (4). However, there are few reports about IgA production induced by dietary antigens. In the present study, we have shown that dietary antigens contribute to IgA production and PP responses in the gut.

We found that GF mice had similar numbers of GC B cells and Tfh cells in PPs compared to SPF mice. In contrast to our data, some previous reports have shown that GF mice had reduced numbers of these cells compared to SPF or conventional mice (37, 38). On the other hand, another report has shown results similar to ours (39). A possible reason for the discrepancy among these experiments may be differences in animal housing conditions among laboratories. For example, normal mouse chow frequently contains dead bacteria originating as a contaminate in fish powder used as a chow ingredient. There is also variation in the amount of bacterial components between different types of diets, which can cause diverse outcomes in the status of immune responses (40). Additionally, in several studies, a chemically defined, water soluble, low molecular weight diet, which resembles the antigen-free diet in our study, was used to maintain the GF mice (41, 42). Thus, these dietary differences may have contributed to the observed differences in the PP responses in GF mice in the different studies. Another possibility is that variations in the microbiota of SPF mice among different animal facilities caused the difference in PP responses, which resulted in the observed changes in GC responses between SPF mice and GF mice.

As an underlying mechanism for the induction of IgA by dietary antigens, Foxp3^+^ T cells induced by dietary antigens could convert into Tfh cells to induce IgA responses in PPs (4, 27). Indeed, Nrp-1^−^Rorgt^−^Foxp3^+^ T cells in PPs of GF-AF mice were reduced compared to GF mice (Supplemental Figure 4A). On the other hand, Th17 cells, which could also convert into Tfh cells, were almost absent in GF mice (Supplemental Figure 4B). Importantly, addition of BSA to the AF diet used in out studies restored the number of GC B cells (Supplemental Figure 3B), further corroborating the contribution of dietary antigens to the IgA response.

ILFs are one of the important IgA inductive sites in the gut. The number of ILFs and mILFs are altered by the presence of the microbiota (Figure 4). GF-AF mice and GF mice have similar numbers of ILFs and mILFs, while SPF-AF mice have fewer ILFs and mILFs compared to SPF mice. The decrease in the number of ILFs in SPF-AF mice might due to changes in the microbiota because the fecal microbiota composition in SPF-AF mice was different from that in SPF mice (Supplemental Figures 2C,D), consistent with a previous report (4). Therefore, these data suggest that dietary antigens have an effect on the GC responses in PP, but not on the development of ILFs. These results are consistent with a previous report indicating a role for PPs, but not ILFs, in the induction of antigen-specific response to an orally administered antigen (7).

We focused here on the effect of dietary antigens on protection against *S*. Typhimurium because IgA production is crucial for this protection (43, 44). Our study demonstrated that GF-AF mice were more susceptible to *S*. Typhimurium infection than GF mice, and that this was associated with the reduction of *S*. Typhimurium-specific IgA. Mechanistically, polyreactive natural IgA induced by dietary antigens presumably mediates the entrance of *S*. Typhimurium into PPs, which allows the induction of *S*. Typhimurium-specific IgA to protect against infection by these microbes (45). In addition, the decrease in the frequency of Nrp-1^−^Rorgt^−^Foxp3^+^ T cells in the PPs in GF-AF mice (**Supplemental** Figure 4A) may also be involved in the induction of the antigen-specific IgA response, because Foxp3^+^ T cells induce GC and IgA responses by generating GC Tfh cells (27) and their depletion causes the loss of specific IgA responses (46). The detailed molecular mechanisms for the induction of antigen-specific IgA by Foxp3^+^ T cells have not yet been clarified and further studies are required in order to examine the issue. On the other hand, IFN-γ is known as an important factor for protecting the host from *S*. Typhimurium infection (47). It has been reported that Th1 cells, which are responsible for IFNγ production, are decreased in GF-AF mice (4). Therefore, it is possible that Th1 cells induced by dietary antigens also contribute to protection against *S*. Typhimurium infection.

In conclusion, we here demonstrate that dietary antigens are crucial for efficient IgA production through induction of GC responses in PPs. probably via differentiation of Tfh cells. This in turn contributes to immune protection against oral pathogens such as *S*. Typhimurium.

## Materials and Methods

### Mice

C57BL/6N mice were purchased from CLEA Japan Inc. (Tokyo, Japan). AID^−−/−^ mice were provided by T. Honjo [Kyoto University, Kyoto, Japan; (48)]. The mice were maintained under SPF or GF condition in the animal facilities of Yokohama City University until use in experiments at 9–13 weeks-old. Mice were kept under conventional conditions after oral infection with *S*. Typhimurium. For generating AF mice, mice were fed with an antigen-free diet composed of a liquid diet and an oil diet after weaning at 3–3.5 weeks-old. The method for preparing the antigen-free diet is described elsewhere (4). For some experiments, 1% BSA was added to the antigen-free diet. All animal experiments were performed in accordance with protocols approved by the animal studies committees of Yokohama City University and RIKEN.

### Cell Isolation

Intestinal lamina propria lymphocytes were collected as described previously (49). Briefly, the small and large intestines were opened and washed carefully, after removal of PPs and fat, and incubated with RPMI 1640 medium containing 1 or 2 mM EDTA and 2% FBS for 15 min at 37°C. Tissues were then minced and incubated with RPMI 1640 containing 2% FBS and 1 mg/ml collagenase (Wako) for 15 min three times at 37°C. Immune cells in the digested tissues were separated and collected by Percoll gradient centrifugation. Immune cells from PPs, MLNs, and the spleen were collected by mashing these tissues through a 100 μm mesh. These cells were used for flow cytometry analysis and culture. To detect the production of IL-17A, cells were cultured with 1 μg/ml Ionomycin, 25 ng/ml PMA (Sigma) and 5 μg/ml Brefeldin A (Biolegend) for 4 h before staining and flow cytometry.

### Flow Cytometry Analysis

Cells were stained with Zombie Aqua Fixable Viability Kit (Biolegend) to label living cells and with anti-CD16/CD32 Fc-block antibody (BD Pharmingen) for 15 min at 4°C. The cells were then incubated with surface marker antibodies; GL7 (GL7) from eBioscience or BD Biosciences, B220 (RA3-6B2), CD95 (Jo2), CD62L (MEL-14), CD4 (RM4-5) from eBioscience, CD3 (145-2C11), CD44 (IM7), Neuropilin-1 (3E12), CD45.2 (104), CD19 (6D5), CXCR5 (J252D4) from BioLegend, and IgA (11-44-2) from Southern Biotech. For intracellular staining, cells were fixed and permeabilized using an intracellular staining kit (Foxp3/Transcription Factor Staining Buffer set; Thermo Fisher Scientific) and incubated with the following antibodies; Foxp3 (FJK-16s) from eBioscience, RORγt (Q-31-378) from BD Biosciences, and IL17A (TC11-18H10.1) and IFNγ (XMG1.2) from Biolegend.

**ELISA**. Feces were frozen and dried and then homogenized in PBS contacting a 1 × protease inhibitor cocktail (Roche). The homogenized samples were centrifuged at 14,000 rpm for 5 min. IgA in the fecal supernatants or from plasma was measured using a mouse ELISA Quantification set (Bethyl laboratories, Inc.) according to the manufacturer's protocol. To detect *S*. Typhimurium-specific IgA in feces, plates were incubated with 50 μl of 5 × 10^9^ *S*. Typhimurium cells/ml overnight, then cells were fixed with 0.15% glutaraldehyde in 0.15 M phosphate buffer, pH 7.0 solution for 5 min, followed by incubation with 0.15 M glycine in 15 mM phosphate buffer, pH 7.0 for 5 min. Next, the plates were incubated with 2% BSA for 2 h. After washing, supernatants of feces were added to the wells and incubated for 2 h, and then washed. After this procedure, the plates were incubated with HRP anti-mouse IgA (Bethyl laboratories, Inc.) for 1 h and washed, then TMB solution (Thermo Fisher) was added to the wells for 15 min. The reaction was then stopped by addition of H_2_SO_4_, and the IgA concentration was measured by absorbance at 450 nm based on a standard curve.

### Microbiota Analysis

Fecal samples were collected and DNA was extracted according to a previous study with minor modifications (50, 51). In brief, feces were suspended in TE10 containing 15 mg/ml lysozyme (Wako Pure Chemical Industries) at 37°C for 1 h. Achromopeptidase (Wako Pure Chemical Industries) was then added to a final concentration of 2,000 U/ml and the samples were incubated at 37°C for 30 min. 1% sodium dodecyl sulfate and 1 mg/ml proteinase K (Merck) were added to the samples, which were incubated at 55°C for 1 h. After centrifugation to remove debris, DNA was extracted with a phenol/chloroform/isoamyl alcohol (15:24:1) solution and ethanol and sodium acetate were added for precipitation.

16S rRNA amplicon sequencing on an Illumina Miseq was performed according to Kozich et al. (52). Each reaction mixture contained 0.2 mM deoxyribonucleoside triphosphates, 5 μl of 10X Ex Taq HS buffer (Takara Bio Inc.) 15 pmol of primers for amplification of the V4 variable region (515F to 806R). PCR was conducted as follows; 95°C for 2 min and 25 cycles of 95°C for 20 s, 55°C for 15 s and 72°C for 5 min, then finally 72°C for 10 min. The amplified DNA samples were purified using AMPure XP (Beckman Coulter) and quantified by a Quant-iT PicoGreen ds DNA Assay kit (Thermo Fisher Scientific). Approximately equal amounts of PCR amplicons from each sample were pooled into mixed samples, which were analyzed with a High Sensitivity DNA kit by using a 2100 Bioanalyzer (Agilent Technologies). Real-time PCR for quantification was performed with the mixed samples by using a KAPA Library Quantification kit (Illumina) following the manufacturer's instructions. The mixed samples with 20% denatured PhiX spike-in were sequenced by Miseq using a 500 Cycles kit (Illumina). The QIIME software package was used for taxonomic assignments and estimation of relative abundance of sequencing data (53). UCHIME was performed for chimera checking (54). The operational taxonomic units (OTUs) were defined at 97% similarity, and the OTUs indicating relative abundance of <1% were filtered to remove noise. The OTUs were assigned a taxonomy by comparison to the Greengenes database using RDPclassifier (55, 56).

### Histological Analysis

Samples were fixed in 4% paraformaldehyde in PBS for 2 h on ice followed by treatment with 30% sucrose in PBS overnight, and then embedded in OCT compound. Frozen sections were treated with 1% blocking solution in PBS for 1 h at RT before stained with primary antibody. Armenian hamster anti-CD3ε (145-2C11), rat anti CD45R/B220 (RA3-6B2) conjugated with Alexa647, and rat GL7 conjugated with FITC were used for primary antibodies, and CD3ε staining was detected with Alexa549-conjugated anti Armenian hamster IgG. DAPI (DOJINDO LABORATORIES) was also used for nuclear staining. The sections were analyzed with a fluorescence microscope (Leica DMI6000B, Leica Microsystems).

### ILF Staining and Size Measurement

The following intestinal segments were used for ILF counting; proximal SI, 5 cm piece of SI after removing 1 cm from the pylorus; distal SI, 5 cm piece of SI from 1 cm above of the ileocecal junction; proximal LI, the upper half of the LI; and distal LI, the lower half of the LI. These intestinal tissues were opened and carefully washed in TBS. The tissues were then incubated in HBSS buffer containing 5 mM EDTA and 10% FCS for 30 min at 37°C twice while shaking, washed in TBS, and fixed with 10% formalin in TBS for 1 h at 4°C. After washing in TBS for three times, the tissues were incubated with 0.3% H_2_O_2_ for 15 min at room temperature, washed in TBS, and then incubated with solution A containing 50 mM Tris (pH 7.2), 150 mM NaCl, 0.6% Triton-X and 0.1% BSA for 1 h at 4°C. The tissues were then treated with blocking solution (Block ace; DS Pharma Biomedical Co., Ltd) for 1 h at 4°C, incubated with anti-mouse B220 antibody conjugated with biotin (RA-6B2) in solution A overnight at 4°C, and washed. Subsequently, the tissues were incubated with ABC reagent (VECTASTAIN Elite ABC Standard kit, VECTOR) diluted with solution A for 2.5 h at room temperature. After washing, the tissues were reacted with DAB reagent diluted with solution A to develop color in the B220 positive area and were washed immediately afterwards. The tissues were dehydrated by incubating with 95% ethanol twice, then replaced by xylene to be mounted on slides. The specimens were observed under a microscope (SZX16, OLYMPUS) to count the ILFs and measure the size of ILFs with a software (DP2-BSW, OLYMPUS). ILFs with aggregates of B220^+^ cells ≥50,000 μm^2^ were defined as a mILF (30–32).

### Counts of PP Follicles and Measurement of PP Sizes

For visualizing follicles in PPs and measuring the size of PPs along the small intestinal wall, intact SI was incubated in 3% acetic acid for 15 min at room temperature thereby dehydrating the specimens. The number of follicles in the specimens were counted under a microscope (SZX16, OLYMPUS), and the sizes of PPs were measured with software (DP2-BSW, OLYMPUS).

### *S*. Typhimurium Infection

Mice were infected with Δ*aroA S*. Typhimurium UF20 (5 × 10^9^ CFU mouse; provided by H. Matsui, Kitasato University, Tokyo, Japan; 35) by oral administration. Fecal samples were collected at pre-administration, and at 1 and 2 weeks after administration and treated and analyzed for *S*. Typhimurium-specific IgA by ELISA as described above. Two weeks after administration of Δ*aroA S*. Typhimurium, mice were infected with the virulent *S*. Typhimurium χ3306 [5 × 10^9^ CFU/mouse; provided by H. Matsui; (57)] by oral administration and Kaplan-Meier survival curves were determined.

### Statistical Analysis

Data are expressed as mean ± SD. Welch's *t*-test or one-way ANOVA with Tukey *post-hoc* test were used in figures other than Figure 5. In Figure 5, survival curves analyzed with a Log rank test and the others were analyzed with two-way ANOVA with Bonferroni *post-hoc* test. *P* < 0.05 were considered statistically significant.

## Ethics Statement

All animal experiments were performed in accordance with protocols approved by the animal studies committees of Yokohama City University.

## Author's Note

We dedicate this article to CS, who passed away during the development of this article.

## Author Contributions

HO, SH, TS, TKan, and NS-T conceived the project and designed the experiments. TKat performed the microbiota analysis. SH performed all the other the experiments. YT, TS, MT, and NT supported the preparation of antigen-free mice. NT supported the infection experiment. KK and CS provided a method for preparing the antigen-free diet. SH, TS, TKan, and HO wrote the manuscript. TS, TKan, NS-T, and HO discussed the results and supervised SH.

### Conflict of Interest

The authors declare that the research was conducted in the absence of any commercial or financial relationships that could be construed as a potential conflict of interest.

## Supplementary Material

Supplemental Figure 1 GF-AF mice in this study have a similar phenotype to those in a previous report. **(A)** The levels of serum IgA in SPF (*n* = 5), GF (*n* = 9), and GF-AF (*n* = 8) mice was measured by ELISA. Data are pooled from at least two independent experiments. **(B)** Leukocytes numbers in SI-LP. (SPF; *n* = 7, GF; *n* = 8 and GF-AF; *n* = 7). **(C)** Leukocytes numbers in MLN. (SPF; *n* = 7, GF; *n* = 8 and GF-AF; *n* = 8). **(D)** Leukocytes numbers in spleen. (SPF; *n* = 6, GF; *n* = 6 and GF-AF; *n* = 6). **(E)** Leukocytes numbers in LI-LP. (SPF; *n* = 7, GF; *n* = 8 and GF-AF; *n* = 7). **(F)** IgA concentration in feces from SPF, GF, and GF-AF mice. **(G,H)** The absolute numbers of B220-IgA+ IgA-producing plasma cells in SI-LP **(G)** and LI-LP **(H)** of SPF (*n* = 6), GF (*n* = 6), and GF-AF mice (*n* = 3). **(A–E)** Data are pooled from at least three independent experiments. Data are mean ± SD. One-way ANOVA with Tukey's *post-hoc* test was performed for statistical analysis. ^*^*p* < 0.05, ^**^*p* < 0.01, ^***^*p* < 0.001, ^****^*p* < 0.0001.

Supplementary Figure 2 IgA production and changes in the fecal microbiota composition of SPF-AF mice. **(A)** IgA concentration in feces from SPF (*n* = 6) and SPF-AF mice (*n* = 6). Data are pooled from two independent experiments. **(B)** Representative flow cytometry plots of IgA vs. B220 on CD3^−^ lymphocytes of SI-LP and LI-LP of SPF and SPF-AF mice (left), with the absolute numbers of B220^−^IgA^+^ IgA-producing plasma cells (right). **(C,D)** Microbiota compositions of SPF mice (*n* = 5) and SPF-AF (*n* = 5) mice are shown at phylum level **(C)** and genus level **(D)**. Data are presented as mean ± SD and Welch's *t*-test was used for statistical analysis **(A,B)**. ^**^*p* < 0.01, ^***^*p* < 0.001, ^****^*p* < 0.0001.

Supplementary Figure 3 Dietary antigens affect GC B cells and Tfh cells in PP and MLN. **(A,B)** The number of leukocytes **(A)** and GC B cells **(B)** in PP of GF-AF mice and GF-AF mice fed AF diet supplemented with 1% BSA. **(C,D)** The number of GC B (B220^+^CD19^+^ Fas^+^GL7^+^) cells **(C)** and Tfh (CD19^−^CD3^+^CD4^+^CXCR5^+^PD-1^+^) cells **(D)** in PP of SPF (*n* = 4 or 6) and SPF-AF (*n* = 4 or 6) mice. Data are pooled from at least two independent experiments. **(E,F)** The number of GC B cells **(E)** and Tfh cells **(F)** in MLN of SPF (*n* = 7 or 8), GF (*n* = 8 or 9), and GF-AF (*n* = 8 or 9) mice. Data are pooled from at least two independent experiments. All data are mean ± SD. Welch's *t*-test was used for statistical analysis **(A–D)**. One-way ANOVA with Tukey's *post-hoc* test was performed for statistical analysis **(E,F)**. ^*^*p* < 0.05, ^**^*p* < 0.01, ^****^*p* < 0.0001.

Supplementary Figure 4 Nrp-1^−^RORγt^−^ pTreg cells in PP are reduced in GF-AF mice. **(A)** The number of Neuropilin-1^low^ RORγt^−^ Foxp3^+^ CD4 T cells in PP of SPF (*n* = 4), GF (*n* = 4), and GF-AF (*n* = 5) mice. Data are representative of two independent experiments. **(B)** The number of IL-17A producing CD4 T cells in PP of SPF (*n* = 4), GF (*n* = 3), and GF-AF (*n* = 3) mice. Data are pooled from two independent experiments. All data are mean ± SD. One-way ANOVA with Tukey's *post-hoc* test was performed for statistical analysis. ^*^*p* < 0.05, ^**^*p* < 0.01, ^****^*p* < 0.0001.

Supplementary Figure 5 The development and maturation of ILF are altered by dietary antigen through the microbiota in some parts of the intestine. **(A–D)** Total ILF numbers; **(E–H)** Mature ILF numbers in SPF and SPF-AF mice. Mature ILFs were counted by measuring the size of the B220+ area, and if ≥50,000 μm^2^, the ILFs were characterized as “mature.” The numbers of total and mature ILF were counted in the following parts of the mouse intestine; **(A,E)** Proximal SI. **(B,F)** Distal SI. **(C,G)** Upper half of LI. **(D,H)** Lower half of LI. The intestinal regions were defined as described in the Materials and Methods section. Data are pooled from two independent experiments (*n* = 4). Mean ± SD. are shown. Welch's *t*-test was used for statistical analysis. ^**^*p* < 0.01.

## Abbreviations

AID: activation-induced cytidine deaminase
AF: antigen-free
FAE: follicle-associated epithelium
GALT: gut associated lymphoid tissue
GC: germinal center
GF: germ-free
GF-AF: germ-free antigen-free
ILFs: isolated lymphoid follicles
small intestine: SI
large intestine: LI
lamina propria: LP
mILFs: mature isolated lymphoid follicles
MLN: mesenteric lymph node
PPs: Peyer's patches
SPF: specific pathogen free
SPF-AF: specific pathogen free—antigen free
Tfh: follicular helper T
Th17: T-helper 17
Treg cells: regulatory T cells.
