# Supplementary figures and images for: Dietary Antigens Induce Germinal Center Responses in Peyer's Patches and Antigen-Specific IgA Production
Source: Front Immunol. 2019 Oct 15;10:2432. doi: 10.3389/fimmu.2019.02432 (PMC6803481; doi:10.3389/fimmu.2019.02432)

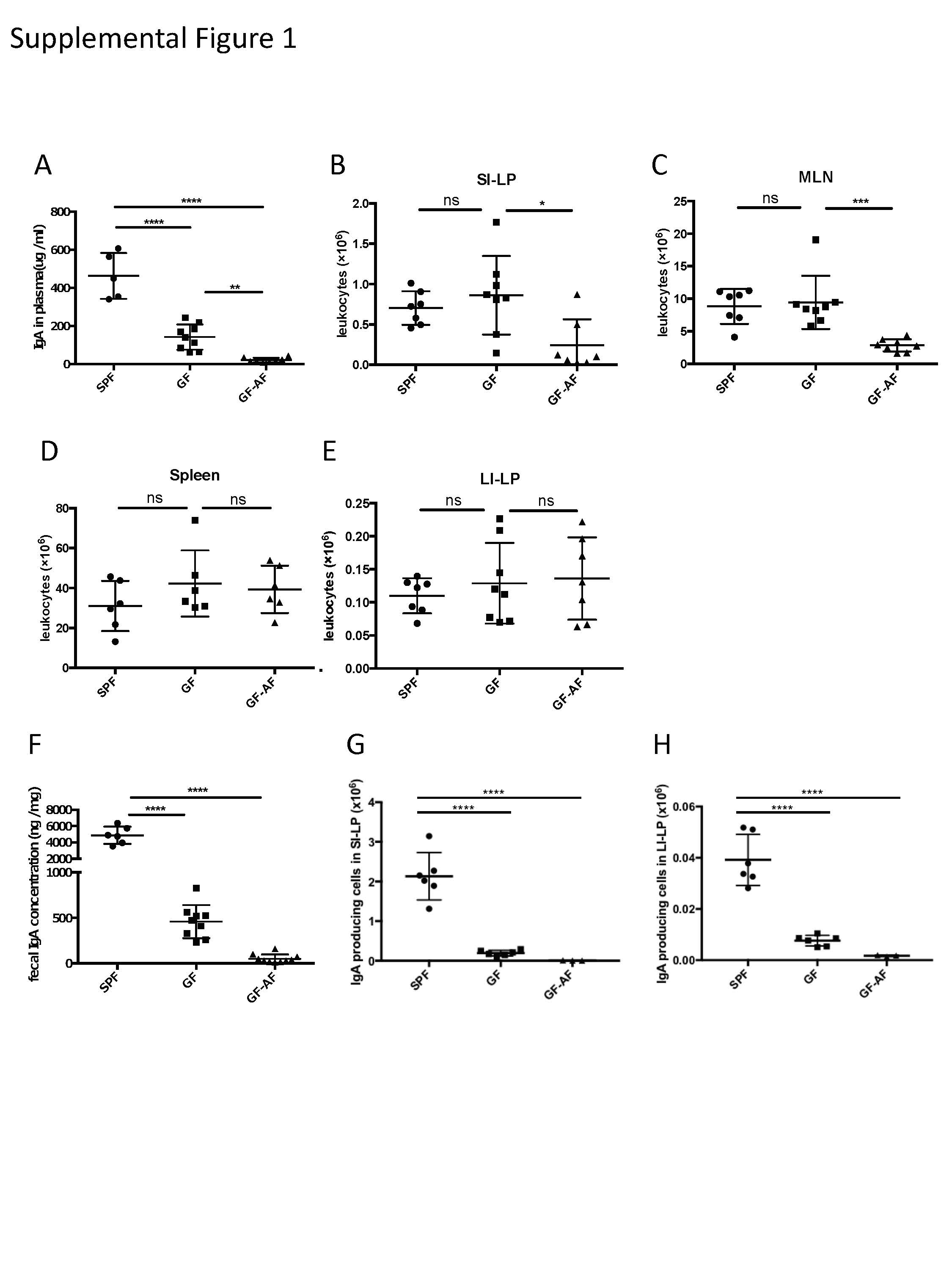

Supplement: Supplemental Figure 1 — GF-AF mice in this study have a similar phenotype to those in a previous report. (A) The levels of serum IgA in SPF (n = 5), GF (n = 9), and GF-AF (n = 8) mice was measured by ELISA. Data are pooled from at least two independent experiments. (B) Leukocytes numbers in SI-LP. (SPF; n = 7, GF; n = 8 and GF-AF; n = 7). (C) Leukocytes numbers in MLN. (SPF; n = 7, GF; n = 8 and GF-AF; n = 8). (D) Leukocytes numbers in spleen. (SPF; n = 6, GF; n = 6 and GF-AF; n = 6). (E) Leukocytes numbers in LI-LP. (SPF; n = 7, GF; n = 8 and GF-AF; n = 7). (F) IgA concentration in feces from SPF, GF, and GF-AF mice. (G,H) The absolute numbers of B220-IgA+ IgA-producing plasma cells in SI-LP (G) and LI-LP (H) of SPF (n = 6), GF (n = 6), and GF-AF mice (n = 3). (A–E) Data are pooled from at least three independent experiments. Data are mean ± SD. One-way ANOVA with Tukey's post-hoc test was performed for statistical analysis. *p < 0.05, **p < 0.01, ***p < 0.001, ****p < 0.0001. [file Image_1.jpeg]

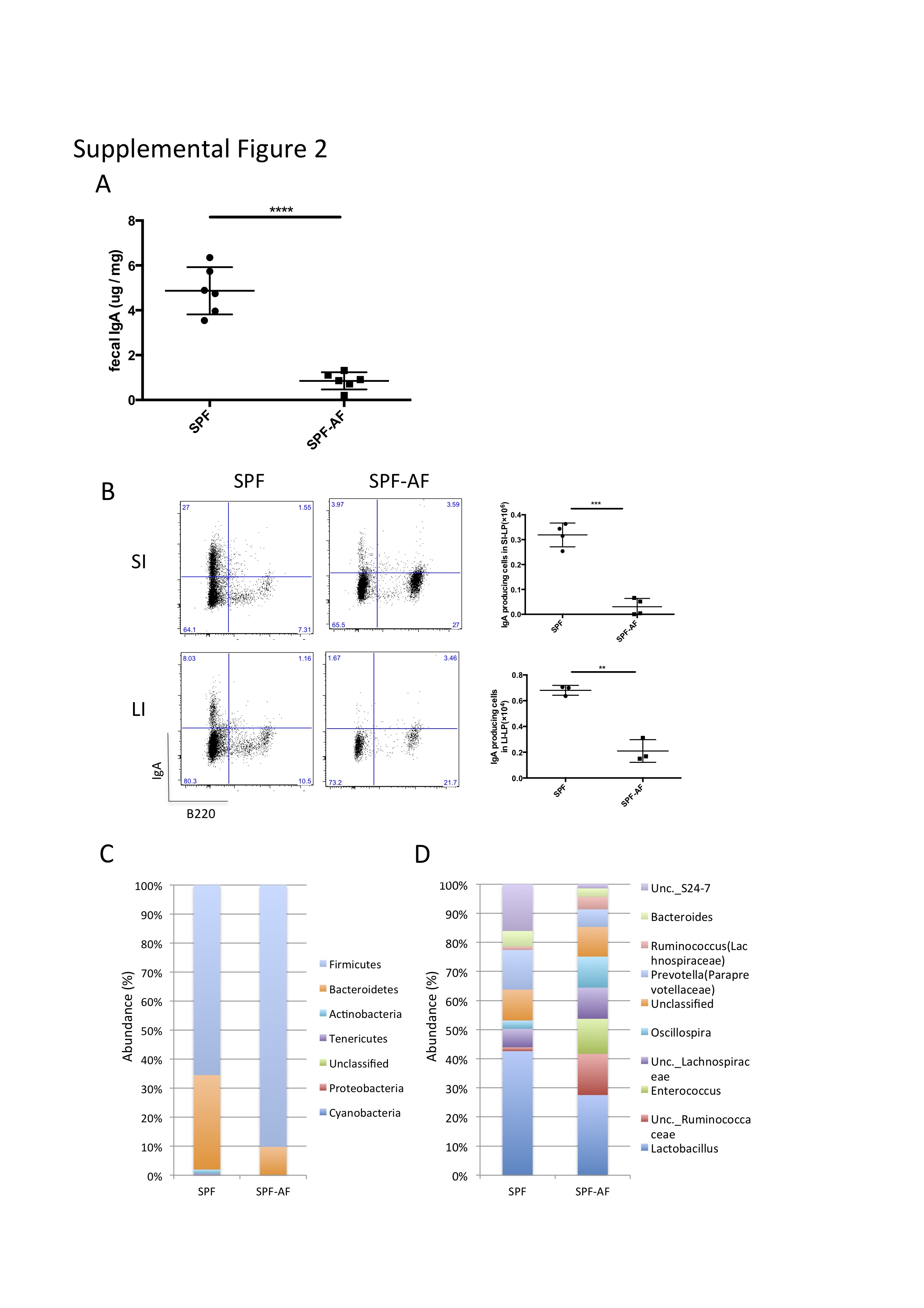

Supplement: Supplementary Figure 2 — IgA production and changes in the fecal microbiota composition of SPF-AF mice. (A) IgA concentration in feces from SPF (n = 6) and SPF-AF mice (n = 6). Data are pooled from two independent experiments. (B) Representative flow cytometry plots of IgA vs. B220 on CD3− lymphocytes of SI-LP and LI-LP of SPF and SPF-AF mice (left), with the absolute numbers of B220−IgA+ IgA-producing plasma cells (right). (C,D) Microbiota compositions of SPF mice (n = 5) and SPF-AF (n = 5) mice are shown at phylum level (C) and genus level (D). Data are presented as mean ± SD and Welch's t-test was used for statistical analysis (A,B). **p < 0.01, ***p < 0.001, ****p < 0.0001. [file Image_2.jpeg]

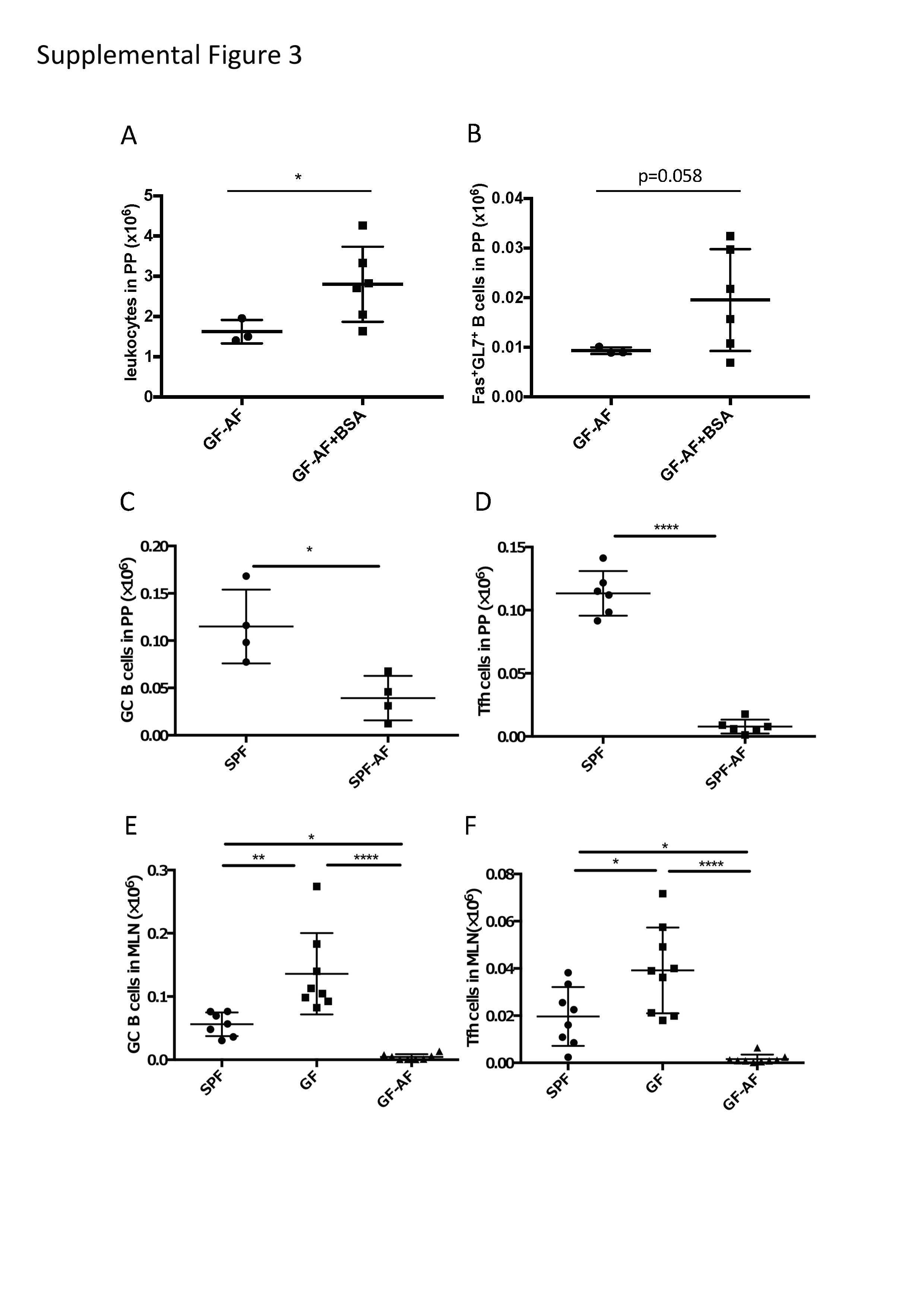

Supplement: Supplementary Figure 3 — Dietary antigens affect GC B cells and Tfh cells in PP and MLN. (A,B) The number of leukocytes (A) and GC B cells (B) in PP of GF-AF mice and GF-AF mice fed AF diet supplemented with 1% BSA. (C,D) The number of GC B (B220+CD19+ Fas+GL7+) cells (C) and Tfh (CD19−CD3+CD4+CXCR5+PD-1+) cells (D) in PP of SPF (n = 4 or 6) and SPF-AF (n = 4 or 6) mice. Data are pooled from at least two independent experiments. (E,F) The number of GC B cells (E) and Tfh cells (F) in MLN of SPF (n = 7 or 8), GF (n = 8 or 9), and GF-AF (n = 8 or 9) mice. Data are pooled from at least two independent experiments. All data are mean ± SD. Welch's t-test was used for statistical analysis (A–D). One-way ANOVA with Tukey's post-hoc test was performed for statistical analysis (E,F). *p < 0.05, **p < 0.01, ****p < 0.0001. [file Image_3.jpeg]

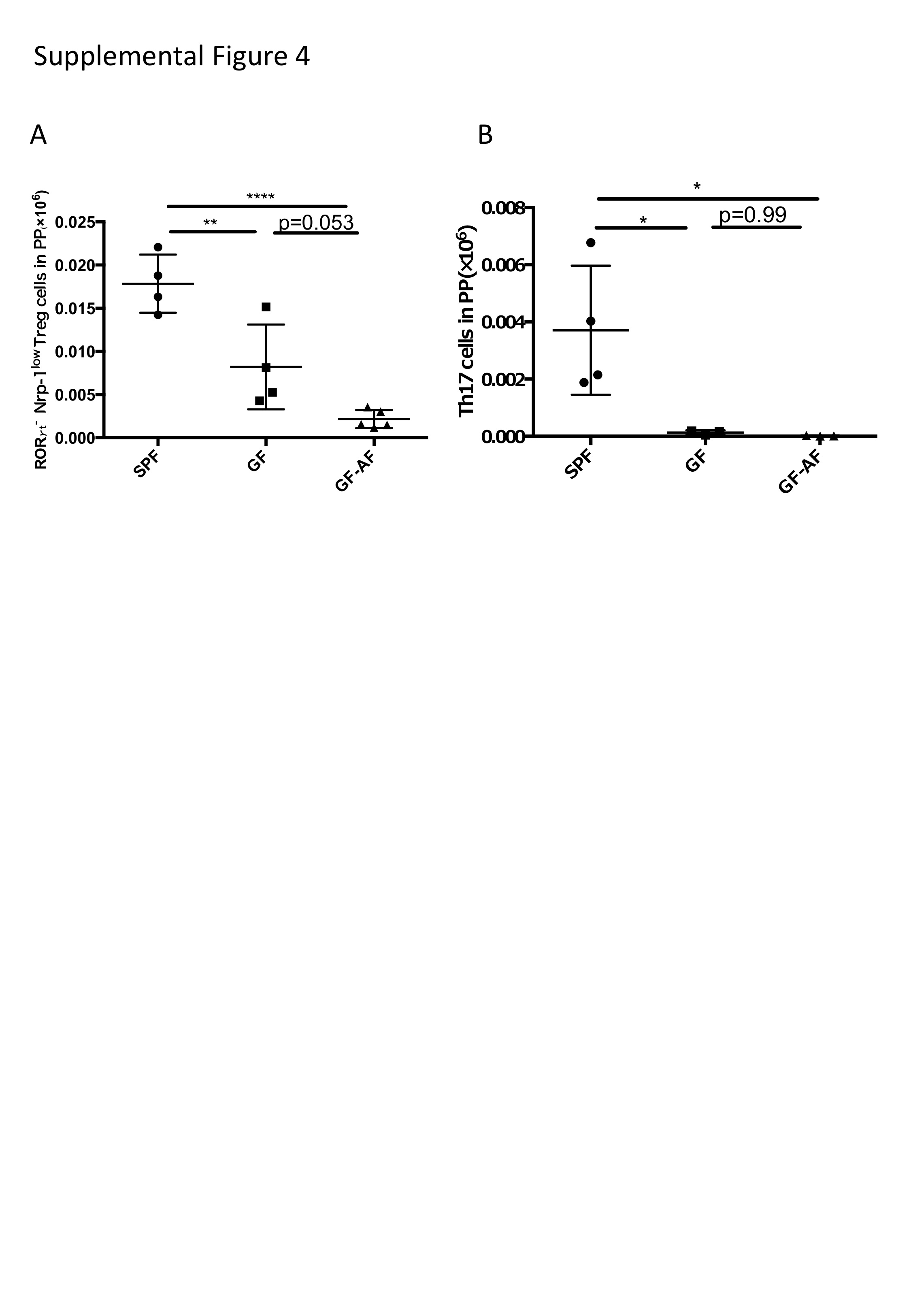

Supplement: Supplementary Figure 4 — Nrp-1−RORγt− pTreg cells in PP are reduced in GF-AF mice. (A) The number of Neuropilin-1low RORγt− Foxp3+ CD4 T cells in PP of SPF (n = 4), GF (n = 4), and GF-AF (n = 5) mice. Data are representative of two independent experiments. (B) The number of IL-17A producing CD4 T cells in PP of SPF (n = 4), GF (n = 3), and GF-AF (n = 3) mice. Data are pooled from two independent experiments. All data are mean ± SD. One-way ANOVA with Tukey's post-hoc test was performed for statistical analysis. *p < 0.05, **p < 0.01, ****p < 0.0001. [file Image_4.jpeg]

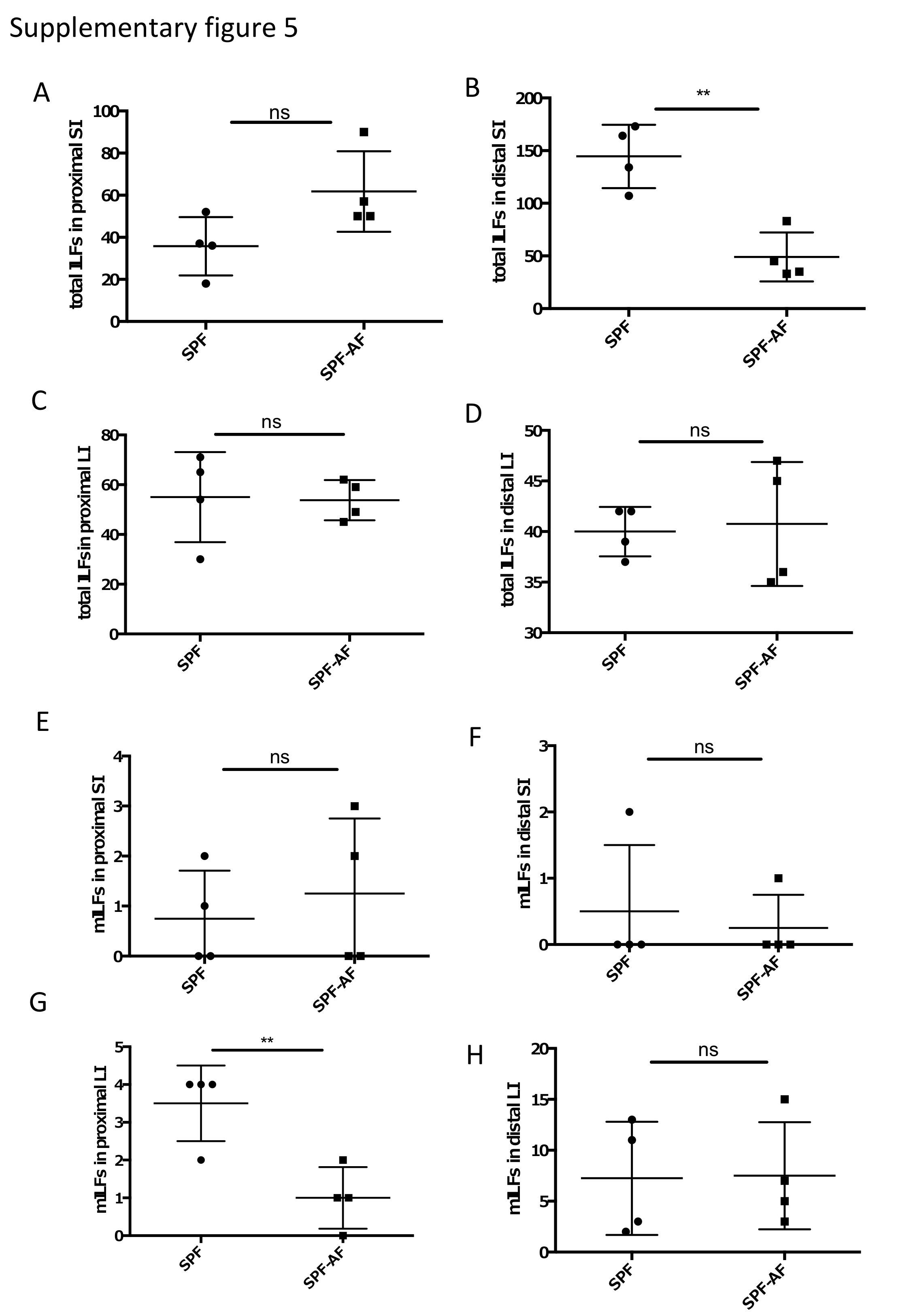

Supplement: Supplementary Figure 5 — The development and maturation of ILF are altered by dietary antigen through the microbiota in some parts of the intestine. (A–D) Total ILF numbers; (E–H) Mature ILF numbers in SPF and SPF-AF mice. Mature ILFs were counted by measuring the size of the B220+ area, and if ≥50,000 μm2, the ILFs were characterized as “mature.” The numbers of total and mature ILF were counted in the following parts of the mouse intestine; (A,E) Proximal SI. (B,F) Distal SI. (C,G) Upper half of LI. (D,H) Lower half of LI. The intestinal regions were defined as described in the Materials and Methods section. Data are pooled from two independent experiments (n = 4). Mean ± SD. are shown. Welch's t-test was used for statistical analysis. **p < 0.01. [file Image_5.jpeg]
